# Supplementary material for: Structure and mechanism of a Hypr GGDEF enzyme that activates cGAMP signaling to control extracellular metal respiration
Source: eLife. 2019 Apr 9;8:e43959. doi: 10.7554/eLife.43959 (PMC6456294; doi:10.7554/eLife.43959)
Supplement: Supplementary file 2. — Primers are listed from 5’ - 3’ [file elife-43959-supp2.docx]

**Table S1. Primers used in this study**

| Sequence 5’ – 3’ | Purpose |
| --- | --- |
| TCT TCG AGT GTG AAG ACC ATT GCG GTC ATT AAG TTT CCT C | cGAMP-nanoluc fusion in pTOPO2.1 |
| GAG GAA ACT TAA TGA CCG CAA TGG TCT TCA CAC TCG AAG ATT | cGAMP -nanoluc fusion in pTOPO2.1 |
| GAG AGA GCT AGC TTA CGC CAG AAT GCG TTC GC | cGAMP -nanoluc fusion in pTOPO2.1 |
| GAG AGG CGC GCC CCG GTT ACC CTT CGG ATG AC | cGAMP -nanoluc fusion in pTOPO2.1 |
| GGT GAA TTG CTG ACT ATA GAA CTA ACA AAC | cdiG-nanoluc fusion in pTOPO2.1 |
| TCT ATA GTC AGC AAT TCA CCG ATA TCG ACA ATA CTA AAC CAT CC | cdiG-nanoluc fusion in pTOPO2.1 |
| AAC GAT CCG GCA GCC CGG GCA AAA AAG AAA TGC CGG GAC | cdiG-nanoluc fusion in pTOPO2.1 |
| GCC CGG GCT GCC GGA TC | cdiG-nanoluc fusion in pTOPO2.1 |
| ACG TGA CAT ATG GAA CGG ATT CTC GTT GTC GAA G | GSU1658 in pRK2-Geo2 F |
| ACT GCT GCT AGC CAC ACT CAA GGT CAA CGG ATT GC | GSU1658 in pRK2-Geo2 R |
| CAG GGC GCG CCT CGT TGA CAA GTG GAC CGC ATT ATC TGA TAG TCT TGG GAA GAC TTG CCA | GSU1658 native promoter F |
| GAC AAC GAG AAT CCG TTC CAT GCA CAA CTC CCG GGG TAG TGG CAA GTC TTC CCA AGA CTA | GSU1658 native promoter R |
| TCT ATA GTC AGC AAT TCA CCG ATA TCG ACA ATG GCA AAC CAT CC | GSU1658 R393A variant |
| GAC CGA GCA TCA CGA ATT GTG ATA TTT CGG CAT CCC GG | GSU1658 R393A variant |
| AAA AAA GAA ATG CCG GGA CCG AGC ATC ACG AAT TGT | GSU1658 R393A variant |
| ACG TGA CAT ATG GCG ATG ACA GCC CTC GTA A | GSU3376 in pRK2-Geo2 F |
| ACT GCT GCT AGC CCA TCT GTT ATG TCG AGC CTG ACA | GSU3376 in pRK2-Geo2 R |
| AGT CGT CTA GAC GAC TTC TTG TGC CTG CTC TCT GG | GSU1658 deletion |
| CGT TGC CCG GAA TAC ACG GTT GAC AAC GAG AAT CCG TTC CAT GCA | GSU1658 deletion |
| TGC ATG GAA CGG ATT CTC GTT GTC AAC CGT GTA TTC CGG GCA ACG | GSU1658 deletion |
| GAGA AGATCT TATATCGATGAACTGACCGG | For primer for Gmet_1914 GGDEF into pET16-MBP-T4lys |
| TTGTTAGCAGCgGccgCACTCGAGCTA ACGAATTGCAGTTGCCCTG | Rev primer for Gmet_1914 GGDEF into pET16-MBP-T4lys |
| GAGA CATATG AATATATTTGAAATGTTACGTATAGATCAAGG | For primer for T4Lys-Gmet_1914 GGDEF into pET24a |
| gtggtggtggtggtggtgctcgagACGAATTGCAGTTGCCCTG | Rev primer for T4Lys-Gmet_1914 GGDEF into pET24a |
| GAGACAT ATGCATAATCCGCATGAATCA | For primer for Codon-optimized WspR into pCOLA |
| GAGACTCGAG TCCAGCAGGTGCCGGA | Rev primer for Codon-optimized WspR into pCOLA |
| GAG GAGTTCACCATAATTCTGGTGGAA | Forward primer for round-the-horn mutagenesis of GSU1658 D373E |
| GCCACCGTAGCGAATGATTA | Reverse primer for round-the-horn mutagenesis of GSU1658 D373E |
| CGT CGTTACCTTGACATCTCGCTG | Forward primer for round-the-horn mutagenesis of GSU1658 Y304R |
| GTTGAAGAGTCCCGTGAGTTC | Reverse primer for round-the-horn mutagenesis of GSU1658 Y304R |
| AGT GAAGCATTACGTCAAGTCG | Forward primer for round-the-horn mutagenesis of Codon-optimized WspR D226S |
| ACCCGCTACATGGCCGAAG | Reverse primer for round-the-horn mutagenesis of Codon-optimized WspR D226S |
| GAT GAGTTTGCAATGGTTCTGC | Forward primer for round-the-horn mutagenesis of Codon-optimized WspR E253D |
| TCCACCATAGCGAGCC | Reverse primer for round-the-horn mutagenesis of Codon-optimized WspR E253D |
| ATGCATAATCCGCATGAATCAAAGACGGACCTGGGAGCTCCACTTGACGGAGCCGTGATGGTTTTATTAGTGGACGACCAGGCGATGATCGGTGAGGCGGTCCGCCGTTCTCTGGCTTCTGAAGCGGGCATCGACTTCCATTTTTGCTCCGATCCGCAGCAAGCGGTAGCGGTAGCCAATCAAATTAAGCCCACGGTTATCCTGCAGGATCTGGTCATGCCTGGCGTGGATGGGCTGACATTGTTAGCAGCTTATCGCGGAAACCCTGCAACACGCGACATTCCGATCATTGTGCTGAGTACCAAGGAGGAACCCACTGTTAAGTCAGCTGCATTTGCAGCCGGGGCGAATGACTACCTGGTCAAACTTCCAGATGCGATCGAATTAGTTGCTCGCATCCGCTACCACAGTCGCAGCTACATCGCGCTTCAGCAACGCGATGAAGCCTACCGCGCCTTGCGCGAATCCCAGCAGCAGCTTCTTGAAACGAACCTGGTTTTGCAGCGTCTGATGAACTCCGACGGTTTAACGGGTTTGTCTAATCGCCGTCATTTTGATGAATACTTAGAGATGGAATGGCGTCGTAGTTTGCGTGAACAATCTCAGTTGTCATTACTTATGATCGACGTCGACTACTTTAAATCGTACAACGATACCTTCGGCCATGTAGCGGGTGACGAAGCATTACGTCAAGTCGCTGGCGCGATCCGTGAAGGGTGCTCCCGTTCTTCTGACCTTGCGGCTCGCTATGGTGGAGAGGAGTTTGCAATGGTTCTGCCTGGGACATCACCGGGGGGCGCTCGCCTGTTGGCTGAGAAAGTGCGTCGCACGGTGGAAAGTTTGCAGATCTCGCATGATCAACCGCGTCCAGGCTCGCATTTAACGGTGTCGATCGGCGTATCCACCTTGGTTCCTGGAGGTGGAGGCCAGACCTTTCGCGTTTTGATCGAAATGGCTGACCAGGCATTATACCAGGCCAAAAATAATGGACGTAATCAGGTGGGATTGATGGAACAACCAGTACCTCCGGCACCTGCTGGA | Codon optimized WspR |
